# An Investigation of the Risk Factors Associated With Anti-Tuberculosis Drug-Induced Liver Injury or Abnormal Liver Functioning in 757 Patients With Pulmonary Tuberculosis

**DOI:** 10.3389/fphar.2021.708522

**Published:** 2021-11-08

**Authors:** Tao Zhong, Yuzheng Fan, Xiao-Li Dong, Xujun Guo, Ka Hing Wong, Wing-tak Wong, Daihai He, Shengyuan Liu

**Affiliations:** ^1^ Department of Tuberculosis Control and Prevention, Shenzhen Nanshan Center for Chronic Disease Control, Shenzhen, China; ^2^ Research Institute for Future Food, The Hong Kong Polytechnic University, Kowloon, Hong Kong SAR, China; ^3^ Department of Applied Biology and Chemical Technology, The Hong Kong Polytechnic University, Kowloon, Hong Kong SAR, China; ^4^ Department of Applied Mathematics, The Hong Kong Polytechnic University, Kowloon, Hong Kong SAR, China; ^5^ Hong Kong Polytechnic University Shenzhen Research Institute, Shenzhen, China

**Keywords:** prescription scheme, risk factor, anti-tuberculosis drug-induced liver injury, pulmonary tuberculosis, China

## Abstract

**Objectives:** To identify the risk factors associated with anti-tuberculosis drug-induced liver injury (AT-DILI) or abnormal living functioning from 757 patients with pulmonary tuberculosis (TB) registered at Nanshan Center for Chronic Disease Control (Nanshan CCDC), Shenzhen, Guangdong Province, China.

**Design and methods:** We identified 757 TB patients who met our inclusion criteria by screening the Hospital Information System (HIS) at Nanshan CCDC. Next, we identified positive cases of AT-DILI or abnormal liver functioning based on results of the first-time liver function tests (LFTs) after taking anti-TB drugs. The χ2 test was used to relate the positive rate with a variety of factors. A logistic regression model was also used to identify statistically significant risk factors.

**Results:** Of the 757 patients, the positive rate of AT-DILI or abnormal liver functioning was 37.9% (287/757). Univariate analysis revealed that the positive rate was 42.91% (212/494) for males and 28.52% (75/263) for females. The positive rate was significantly higher in males (*p* <0.001). Patients with an annual income of 9,231–13,845 USD had a significantly higher positive rate (67.35%; 33/49) than those with an income of 1,540–4616 USD (37.97%; 30/79) (*p* = 0.022). The most frequent prescription regime among positive cases was a 2 months supply of fixed dose combination Ethambutol Hydrochloride, Pyrazinamide, Rifampicin and Isoniazid Tablets (Ⅱ) 450 mg) followed by a 4 months supply of fixed dose combination Rifampin and Isoniazid Capsules (2FDC-HRZE half/4FDC-HR) at 56.03% (144/257). The least frequent prescription regime was a 2 months supply of fixed dose combination Rifampin, Isoniazid and Pyrazinamide Capsules with Ethambutol independently followed by a 4 months supply of fixed dose combination Rifampin and Isoniazid Capsules (2FDC-HRZ + EMB/4FDC-HR) at 24.27% (25/103). The difference between these two different regimes was significant (*p* = 0.022). With an increase in the duration of medication, patients under various prescription regimes all showed a gradual increase in the positive rate of AT-DILI or abnormal liver functioning.

**Conclusion:** We identified several risk factors for the occurrence of AT-DILI or abnormal liver functioning, including gender, annual income, prescription regime, dosage, and treatment time.

## 1 Introduction

Pulmonary Tuberculosis (TB) is a chronic infectious disease that predominantly attacks the lungs and seriously endangers human health. TB is caused by *Mycobacterium tuberculosis*, a bacterium that is spread throughout populations *via* coughs and sneezes. It is critical that we treat TB in an effective manner if we are to reduce morbidity, mortality, and to control epidemics. The treatment of TB includes the oral administration of four different drugs over a period of 6 months. However, both the International Union Against Tuberculosis and Lung Diseases (IUATLD) and the World Health Organization (WHO) recommend prescribers to use fixed-dose combinations (FDCs) in which the four drugs are combined in one tablet (Girling et al., 1988). The application of FDCs can simplify prescriptions, reduce drug abuse, improve patient compliance, and effectively prevent the rising trend of TB drug resistance caused by irrational and irregular treatment (World Health Organization, 1994). The WHO also recommends short courses of FDCs lasting 6–8 months; this strategy has also been proven to be effective for the treatment of TB. However, in practice, studies also showed that some patients could not complete the entire course of treatment, thus leading to a series of adverse effects on epidemic control and even the emergence of multidrug-resistance against TB (The Global Plan to Stop T, 2006; Zigonl et al., 2006).

Many factors may cause treatment interruptions in patients with TB; one of the most important is anti-tuberculosis drug-induced liver injury (AT-DILI). A previous study reported that 11.90% of all liver injuries were found to be attributed to the use of anti-TB drugs (Xia and Zhan, 2007). Another study, involving 4,577 patients taking FDCs across four cities in China, showed that 10.47% of all patients stopped FDC therapy due to liver injury (Kong and Qiao, 2014). Kabbara et al. (2016) reported that the application of isoniazid as a single drug also caused severe liver injury. Research data from the Chronic Disease Prevention and Control Hospital of Nanshan (Nanshan CDC), Shenzhen, China showed that 27% of TB patients registered for FDC treatment from October 2013 to September 2014 stopped TB medications due to drug-induced liver injury (Zhong, 2016).

Nanshan Center for Chronic Disease Control (Nanshan CCDC) is the only public TB-designated medical institution in Nanshan District, Shenzhen, Guangdong Province of China. The functions of this Institute include the prevention, control, diagnosis, and treatment of TB. However, the Institute only provides outpatient treatment for patients with TB and does not provide treatment for more serious underlying diseases or comorbidities such as liver cirrhosis and immunodeficiency. When patients with TB undergo treatment in this Institute, the medical staff provide consistent follow-up over the entire course of treatment. They also observe and record side effects and urge patients to adhere to their daily medications. Some patients experience discomfort due to abnormal liver function or liver injury, and then proceed to stop the medication or refuse treatment on their own initiative, thus leading to treatment failure. In this study, we investigated the risk factors associated with drug-induced liver injury during the treatment of TB. Providing early interventions to address key risk factors will help to prevent further deterioration in liver function and improve treatment compliance. During this study, we acquired and analyzed a series of data from our Hospital Information System (HIS), including individual patient information, prescription regimes, and liver function tests (LFTs).

## Materials and Methods

### Research Objects and Sample Size

We used our HIS system to identify all patients with TB who were registered and treated in our hospital between 2014 and 2019. For each patient, we acquired a range of information, including age, gender (biological sex), education background, income, body mass index (BMI), prescription regimen, duration and dosage of medication, and liver function results. We also considered comorbidities in any patients receiving treatment for TB, including hepatitis B and diabetes. Cases with confirmed hepatitis B, diabetes, and abnormal liver function prior to treatment were excluded. Finally, 757 patients with TB patients who underwent their first round of liver function tests during treatment with anti-TB drugs were recruited. The data collected in this study were not sufficiently detailed to allow scoring on the Roussel Uclaf Causality Assessment Method (RUCAM) scale. Manual for Diagnosis and Treatment of Adverse Tuberculosis Drug Reactions (2009 Edition) is routinely used for diagnosis and treatment at designated TB medical institutions in China and can be used to classify liver function in patients and define abnormal liver function ( 40 U/L< Alanine aminotransferase (ALT) ≤ 80 U/L ), mild liver injury ( 80 U/L< ALT ≤120 U/L or (and) 40 μmol/L < Total bilirubin (TBIL) ≤ 60 μmol/L ), moderate liver injury ( 120 U/L <ALT ≤ 200 U/L or  60 μmol/L < TBIL ≤ 100 μmol/L or 80 U/L< ALT ≤ 120 U/L and 40 μmol/L < TBIL), or severe liver injury (ALT > 200 U/L or TBIL >100 μmol/L ) diagnostic criteria (Xiao, 2009). We used the outcomes of liver function tests to classify our study subjects. Those with abnormal liver function or liver injury were classified as “positive” while those with normal liver function were classified as “negative”.

### Prescriptions of anti-TB Drugs in Nanshan District of Shenzhen, China

Between 2014 and 2019, eight types of first-line anti-TB drugs were prescribed in Nanshan District. Of these, there were four types of FDCs, including 1) FDC-HRZ (Rifampin, Isoniazid and Pyrazinamide Capsules), 2) FDC-HRZE (Ethambutol Hydrochloride, Pyrazinamide, Rifampicin and Isoniazid Tablets (Ⅱ) 900 mg), 3) FDC-HRZE half (Ethambutol Hydrochloride, Pyrazinamide, Rifampicin and Isoniazid Tablets (Ⅱ) 450 mg), and 4) FDC-HR (Rifampin and Isoniazid Capsules). There were four drugs that were prescribed independently, including 1) Isoniazid (INH), 2) Rifampicin (RFP), 3) Ethambutol (EMB), and 4) Pyrazinamide (PZA). The dosage and usage of these drugs are described in the “Guidelines for the Implementation of China’s Tuberculosis Prevention and Control Plan” (The Disease Control and P, 2008). Four prescription regimes were commonly used in our institute: 1) 2 months of FDC-HRZE-half followed by 4 months of FDC-HR (2 FDC-HRZE-half/4FDC-HR; 2) 2 months of FDC-HRZE followed by 4 months of FDC-HR (2FDC-HRZE/4FDC-HR); 3) 2 months of FDC-HRZ with Ethambutol independently (FDC-HRZ + EMB) followed by 4 months of FDC-HR (2FDC-HRZ + EMB/4FDC-HR); 4) 2 months of HRZE followed by 4 months of HR (2HRZE/4HR). Further details are given in Table 1.

**TABLE 1:** Dosage of first-line anti-tuberculosis drugs for adults.

Drug name (each capsule and content)	Weight range	INH (mg)	Contains RFP (mg)	Contains PZA (mg)	Contains EMB (mg)	Usage
Rifampin Isoniazid and Pyrazinamide Capsules (INH80 mg + RFP120 mg + PZA250 mg)	30–39 kg	240	360	750		Once a day
	40–49 kg	320	480	1,000		
	Above 50 kg	**400** ^a^	**600** ^a^	**1,250** ^a^		
Ethambutol Hydrochloride, Pyrazinamide, Rifampicin and Isoniazid Tablets (Ⅱ)900 mg(INH75 mg + RFP150 mg + PZA400 mg + EMB275 mg)	30–37 kg	150	300	800	550	Once a day
	38–54 kg	225	450	1,200	825	
	55–70 kg	**300** ^a^	**600** ^a^	**1,600** ^a^	**1,100** ^a^	
	Above 71 kg	375	750	2000	1,375	
Ethambutol Hydrochloride, Pyrazinamide, Rifampicin and Isoniazid Tablets (Ⅱ)450 mg (INH37.5 mg + RFP75 mg + PZA200 mg + EMB137.5 mg)	30–37 kg	150	300	800	550	Once a day
	38–54 kg	225	450	1,200	825	
	55–70 kg	**300** ^a^	**600** ^a^	**1,600** ^a^	**1,100** ^a^	
	≥71 kg	375	750	2000	1,375	
Rifampin and Isoniazid Capsules (INH150 mg + RFP300 mg)	≥50 kg	**300** ^a^	**600**			Once a day
Isoniazid (INH)(100 mg)		**300** ^a^				Once a day
Ethambutol (EMB)(250 mg)	<50 kg				750	Once a day
	≥50 kg				**1,000** ^a^	
Rifampin (RFP)(150 mg)	<50 kg		**450** ^a^			Once a day
	≥50 kg		600			
Pyrazinamide (PZA)(250 mg)	30–39 kg			1,000		Twice a day
	40–49 kg			1,250		
	≥50 kg			**1,500** ^a^		

a The usual dose for most patients.

### Statistical Analysis

To remove invalid and defective data, we performed data cleaning on personal information, some of the physiological testing data, and prescription information for TB patients. Valid data were then encoded. TB patients who met the inclusion criteria were then identified by R software. Component proportion ratios, based on the different demographic characteristics of patients, were analyzed with incidence descriptions, and the $\chi^{2}$ test was used to compare incidences. IBM SPSS Statistics for Windows, version 23.0 (IBM Corp., Armonk, N.Y., United States) was used for all statistical analyses. *p* < 0.05 was considered to be statistically significant. An increase in the number of days on which patients with TB took medicine led to a developing trend for the positive incidence of liver injury (as determined by the $\chi^{2}$ test) and was statistically significant (*p* < 0.05). A logistic regression model was also established to identify significant risk factors associated with liver injury in TB; the level of significance was set to α=0.05  .

## Results

### Demographic Characteristics of Positive Cases

A total of TB patients taking anti-TB drugs were included in this study, including 494 males (65.26%) and 263 females (34.74%). The age distribution was as follows: 21–30 years (43.86%), 31–40 years (20.48%), >50 years (17.97%), 41–50 years (11.49%), ≤20 years (6.21%). In total, 636 patients provided information relating to their educational background: college degree or above (43.4%), junior high school or below (32.55%), or high school or technical secondary school (24.06%). In total, 425 patients provided their annual income (USD): 22.59% had an income >13,845 USD, 11.53% had an income between 9,231 and 13,845 USD, 27.76% of patients had an income between 4,615 and 9230 USD, 18.59% had an income between 1540 USD and 4614 USD, and 19.53% had an income of ≤1539 USD (which is the minimum standard of living in Shenzhen). We cut the income into these categories such that each category has a substantial proportion of cases. We obtained height and weight measurements from 627 patients; this allowed us to calculate body mass index. Based on BMI values, the 627 patients were classified as normal (63.32%), thin (25.04%), overweight (10.21%), or obese (1.44%). Further details are shown in Table 2.

**TABLE 2:** Demographic characteristics of TB patients and positive rate distribution.

Feature		Number of cases *N*	percentage(%)	Positive cases	Number of negative cases	Positive rate(%)	*X* ^2^	*p*
Gender(757)	Male	494	65.26	212	282	42.91	15.115	<0.001
	Female	263	34.74	75	188	28.52		
Age(Years)(757)	≤20	47	6.21	12	35	25.53	3.664	0.453
	21–30	332	43.86	128	204	38.55		
	31–40	155	20.48	63	92	40.65		
	41–50	87	11.49	32	55	36.78		
	>50	136	17.97	52	84	38.24		
Education level(636)	Junior high school and below	207	32.55	79	128	38.16	1.355	0.508
	High school or technical secondary school	153	24.06	60	93	39.22		
	College degree and above	276	43.40	119	157	43.12		
Annual Income(USD) (425)	≤1,539	83	19.53	36	47	43.37	**11.404**	**0.022**
	1,540–4,614	79	19.59	30	49	37.97		
	4,615–9,230	118	27.76	56	62	47.46		
	9,231–13,845	49	11.53	33	16	67.35		
	>13,845	96	22.59	48	48	50.00		
BMI(627)	Thin: <18.5	157	25.04	55	102	35.03	5.771	0.123
	Normal: 18.5–23.9	397	63.32	176	221	44.33		
	Overweight: 24–27.9	64	10.21	24	40	37.50		
	Obesity: >=28	9	1.44	2	7	22.22		

Bold values represents that they are statistically significant.

The positive cases were diagnosed based on the first-round liver function test results among TB patients after taking anti-TB drugs. We identified a total of 287 positive patients with AT-DILI or abnormal liver functioning, including 212 males (42.91%) and 75 females (28.52%). The proportion of positive cases was significantly higher in males than in females ( $\chi^{2}=15.115$ , P<0.001 ). The distribution of positive cases in different annual income ranges were as follows: 9,231–13,845 USD (67.35%), >13,845 USD (50%), 4,615–9230 USD (47.46%), ≤1539 USD (43.37%), and 1,549–4614 USD (37.97%); there were significant differences between different income ranges ( $\chi^{2}=11.404$ , P=0.022 ). (see Table 2).

Tests showed that TBIL was normal in all positive cases of AT-DILI or abnormal live functioning and that the highest concentration of ALT was 1179 U/L (occurring in one patient). Abnormal liver functioning cases accounted for 73.86% (210/287) of all positive cases, mild AT-DILI accounted for 13.59% (39/287), and moderate AT-DILI accounted for 4.88% (14/287). Severe AT-DILI accounted for 7.67% (22/287 ) of all positive cases. When considering all 757 patients, 9.9% (75/757) were diagnosed with AT-DILI (Table 3).

**TABLE 3:** First-time liver function test results of all subjects after the start of anti-TB drug treatment.

ALT	Male	Female	Total	Percentage(%)
Abnormal	158	54	212	73.86
Mild liver injury	28	11	39	13.59
Mediate liver injury	8	6	14	4.88
Severe liver injury	18	4	22	7.67
Total	212	75	287	100

### Effects of Drug Prescriptions on Anti-Tuberculosis Drug-Induced Liver Injury or Abnormal Live Functioning

The initial anti-TB treatment scheme generally included an intensive 2 month period followed by another 4 months continuation period. Based on the first-time liver function test results after taking anti-TB drugs, the distribution of positive cases in TB patients under different treatment schemes was as follows in decreasing order: 2FDC-HRZE half/4FDC-HR (56.03%), 2FDC-HRZE/4FDC-HR (31.11%), single-drug combination (2HRZE/4HR) (29.32%), and 2FDC-HRZ + EMB/4FDC-HR (24.27%); there were significant differences between these different drug regimens ( $\chi^{2}=55.391$ , P<0.001 ). Furthermore, pairwise comparisons found that the positive rate in patients taking the 2FDC-HRZE half/4FDC-HR regime (56.03%) was significantly higher than those taking the 2FDC-HRZ + EMB/4FDC-HR regime ( $\chi^{2}=29.778$ , P<0.001 ); 2FDC-HRZE/4FDC-HR regime beats 2FDC-HRZE half/4FDC-HR regime significantly ( $\chi^{2}=16.559$ , P<0.001 ); 2FDC-HRZE/4FDC-HR regime also beats single drug combination regime significantly ( $\chi^{2}=41.128$ , P<0.001 ) (see Table 4).

**TABLE 4:** Different drug prescriptions in TB patients and positive rate distribution.

Prescription regime	samples/N	Positive case	Positive percentage (%)	*X* ^2^	*p*
**Comparison among four schemes**				**55.391**	**0.000**
^a^ **2FDC-HRZ + EMB/4FDC-HR**	103	25	24.27	**29.778**	**0.000**
**2FDC-HRZE half/4FDC-HR** ^a^	257	144	56.03		
^a^ **2FDC-HRZ + EMB/4FDC-HR**	103	25	24.27	1.128	0.288
**2FDC-HRZE/4FDC-HR**	90	28	31.11		
**2FDC-HRZE half/4FDC-HR** ^a^	257	144	56.03	**16.559**	**0.000**
**2FDC-HRZE/4FDC-HR**	90	28	31.11		
**2FDC-HRZE half/4FDC-HR** ^a^	257	144	56.03	**41.128**	**0.000**
**2HRZE/4HR**	307	90	29.32		
**2FDC-HRZE/4FDC-HR** ^a^	90	28	31.11	0.107	0.743
**2HRZE/4HR**	307	90	29.32		
**Total**	757	287	37.91		

Bold values represents that they are statistically significant.

a is the result of pairwise comparison. Pairwise comparison between the four groups, *p* × *α* = 0.05, need to modify the inspection level: α' = *α*/N , *N* = *n*(*n*-1)/2. *N* = 4 × (4–1)/2 = 6, *α*' = 0.05/6 = 0.0083.

### Effects of Duration of Medication on Anti-Tuberculosis Drug-Induced Liver Injury or Abnormal Live Functioning

**FIGURE 1:**
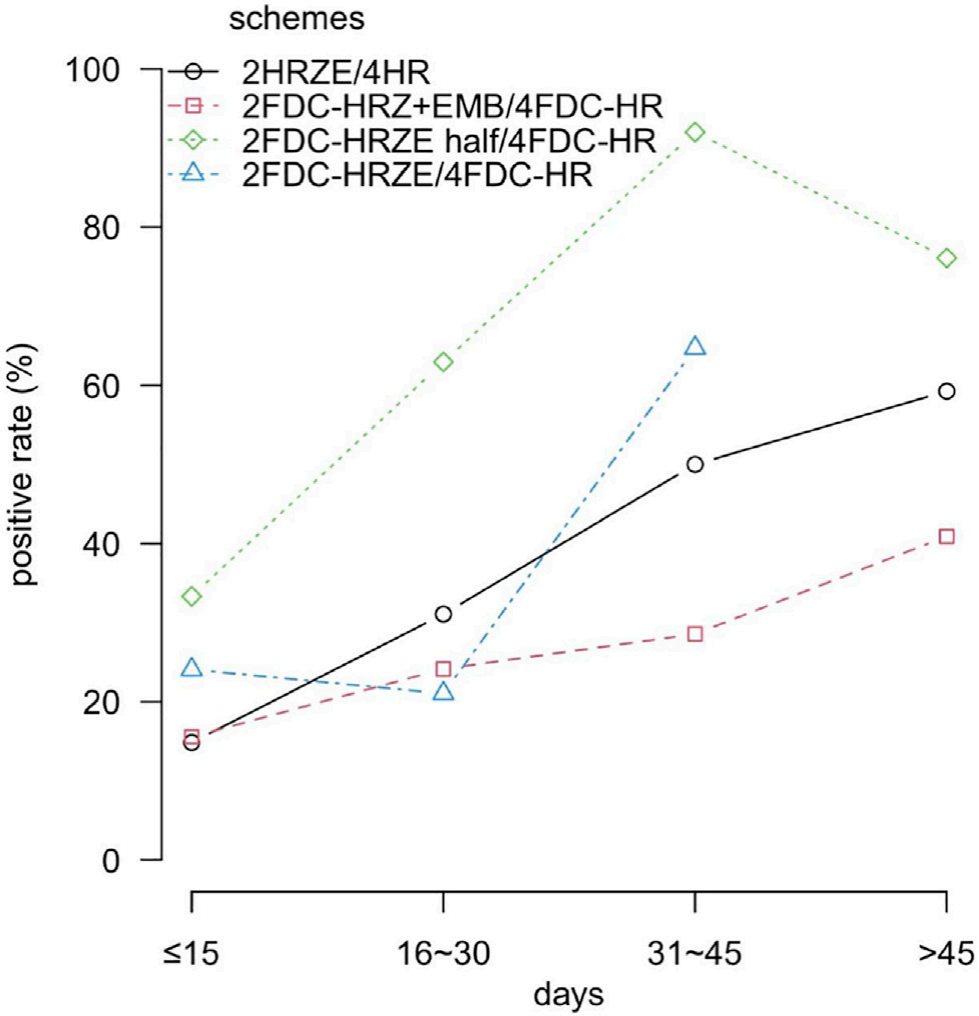
Positive rate versus different prescription regimens and duration of medications.

We divided the number of medication days into four 15 days intervals in the first 2 months intensive period, but four 30 days intervals in the following 4 months continuation period. As shown in Table 5; Figure 1, the positive incidence when taking 2HRZE/4HR for ≤15 days was the lowest (14.84%); the highest incidence was after taking drugs for 61–90 days (62.5%); this difference was significant ( $\chi^{2}=44.158$ , P<0.001 ). As the number of medication days increased, the positive incidence also increased significantly, irrespective of which prescription regime was considered ( $\chi^{2}=39.227$ , P<0.001 ). The positive incidence in patients taking the 2FDC-HRZ + EMB/4FDC-HR regime did not show significant differences when compared across different time intervals $\chi^{2}=5.244$ , P=0.155 ). The patients taking the 2FDC-HRZE/4FDC-HR regime exhibited the lowest positive rate over a period of 16–30 days (21.05%); the highest positive rate occurred when this regime was taken over >30 days (64.71%); these results were significantly different ( $\chi^{2}=11.097$ , P=0.004 ). The positive rate of abnormal liver function increased significantly as the duration of drug treatment increased (*χ* ^2^ = 7.482, *p =* 0.006) (Table 5; Figure 1).

**TABLE 5:** Liver function positive rate versus the duration of anti-TB drug treatment.

Prescription regime	Days	Number of cases N	Composition (%)	Number of positive cases	Number of negative cases	Positive percentage (%)	Rate comparison		Trend *t*est	
							*X* ^2^	*p*	*X* ^2^	*p*
2HRZE/4HR(307)	≤15	155	50.49	23	132	14.84	44.158	0.000	39.227	<0.001
	16–30	74	24.10	23	51	31.08				
	31–45	24	7.82	12	12	50.00				
	46–60	12	3.91	7	5	58.33				
	61–90	8	2.61	5	3	62.50				
	>90^a^	34	11.07	20	14	58.82				
2FDC-HRZ + EMB/4FDC-HR(103)	≤15	45	43.69	7	38	15.56	5.244	0.155	5.141	0.023
	16–30	29	28.16	7	22	24.14				
	31–45	7	6.80	2	5	28.57				
	>45^a^	22	21.36	9	13	40.91				
2FDC-HRZE half/4FDC-HR(257)	≤15	105	40.86	35	70	33.33	51.99	0.000	21.761	<0.001
	16–30	81	31.52	51	30	62.96				
	31–45	25	9.73	23	2	92.00				
	46–60	16	6.23	14	2	87.50				
	61–90	18	7.00	16	2	88.89				
	>90^a^	12	4.67	5	7	41.67				
2FDC-HRZE/4FDC-HR (90)	≤15	54	60.00	13	41	24.07	11.097	0.004	7.482	0.006
	16–30	19	21.11	4	15	21.05				
	>30^a^	17	18.89	11	6	64.71				

a Data are compared after merging.

### Multi-Factorial Analysis of the Risk Factors for Positive Cases

A logistic regression model was established to perform multi-factor analysis with positive status as the dependent variable; the independent variables were those that had been found to be significantly related to positive status: gender, annual income, and medication scheme. The assignment of dependent and independent variables is shown in Table 6. Our analysis showed that male gender ( P=0.011 , odds ratio OR=1.715 ), high annual income ( P=0.024 , OR=1.168 ) and the 2FDC-HRZE half/4FDC-HR medication scheme ( P<0.001 , OR=3.057 ) were all significant risk factors for AT-DILI or abnormal liver functioning. When other variables were controlled and remained changed, we found that male TB patients were 1.715 times more likely to develop AT-DILI than female patients; TB patients with higher incomes were 1.168 times more likely to develop AT-DILI than those with lower incomes; and patients taking the 2FDC-HRZE half/4FDC-HR medication scheme were 3.057 times more likely to develop AT-DILI than those taking the 2HRZE/4HR medication scheme. Detailed results are shown in Table 7.

**TABLE 6:** Independent variable and dependent variable assignment.

Relevant factors	Variable name	Assignment description
Liver function positive	Yes	No = 0, Yes = 1
Gender	X1	Female = 0, Male = 1
Annual income	X2	≤1,539 = 1, 1,540–4,614 = 2
		4,615–9,230 = 3, 9,231–13,845 = 4
		>13,845 = 5
Prescription regime	X3	2FDC-HRZE half/4FDC-HR = 1
		2FDC-HRZ + EMB/4FDC-HR = 2
		2FDC-HRZE/4FDC-HR = 3
		2HRZE/4HR = 4

**TABLE 7:** Multivariate analysis of risk factors of positive cases.

Relevant factors	Reference group	*β*	*S*_*‾x*_	*Wald χ* ^*2*^	*p value*	*OR value*	95%*CI*
Gender							
Male	Female	0.539	0.212	6.484	0.011	1.715	1.132–2.597
Annual Income		0.156	0.069	5.106	0.024	1.168	1.021–1.337
Prescription regime							
2FDC-HRZE half/4FDC-HR	2HRZE/4HR	1.117	0.218	26.289	0.000	3.057	1.994–4.686

## Discussion

Anti-TB drugs or their metabolites may induce allergic reactions in the liver and lead to hepatitis (Qiu, 2012). The occurrence of AT-DILI or abnormal liver functioning may be attributed to drugs and the physiological condition of patients. Risk factors of AT-DILI or abnormal liver functioning include the type and dosage of drugs, the duration of medication, and the combination of medications. The occurrence of AT-DILI or abnormal liver functioning is also associated with age, gender, genetics, the nutritional condition of patients, and the presence of comorbidities. The combination of INH, RFP, EMB, and PZA, also referred to as an FDC, is the standard first line treatment for TB (Huai et al., 2019). However, 0.8–40% of patients worldwide are known to suffer from AT-DILI induced by anti-TB drugs (Chen et al., 2015).

In the present study, our subjects had normal liver function before taking anti-TB drugs but went on to develop abnormal liver function or AT-DILI after taking drugs. Although we did not grade our patients with the RUCAM score, the risk factors used herein (e.g., prescription regimes) were reconfirmed by clinicians during the diagnosis and treatment of patients in designated TB medical institutions in China. Accordingly, clinicians stopped anti-TB drug treatment in most of the AT-DILI patients and placed them on liver protection treatments until their liver function returned to normal. Consequently, we were unable to relate our findings to the RUCAM scale.

Since 2010, patients with TB have been provided with a free FDC therapy plan in Guangdong Province of China. Furthermore, self-paid single-drug treatment is also administered to patients who were not able to take FDCs. In general, we prefer to prescribe adult patients with FDCs if they do not have any other form of complication. However, those with liver dysfunction or other diseases are provided with a single drug. In China, the standard FDC scheme involves 2HRZE/4HR (The Disease Control and P, 2008).

It is well known that hepatitis B and diabetes are significant risk factors for AT-DILI. Consequently, our institute operates enhanced observation and follow-up of TB patients if they are diagnosed with hepatitis B and/or diabetes. Prior to carrying out this study, we compared the positive rate of AT-DILI or abnormal liver functioning between patients with and without these comorbidities and found no statistically significant difference (Table 8). Therefore, we excluded patients with hepatitis B and diabetes.

**TABLE 8:** Liver function test results versus comorbidities among TB patients.

Comorbidities	LFTs negative	LFTs positive	$\chi^{2}$	*p*
Hepatitis B	13	6	2.477	0.29
Diabetes	18	9		
None	239	193		

Our study cohort featured more males than females; furthermore, 82% of our cohort were under 50 years-of-age. The prevalence of TB across all age groups was similar to that reported by our institute for TB epidemic data (Zhong et al., 2018). In this study, age was not identified as a significant risk factor for AT-DILI. This might be because older TB patients are most likely to have complications and were therefore excluded from our study. Furthermore, there is a current trend for TB to affect the younger population. In our study, we found that male TB patients had a higher positive incidence of AT-DILI than female patients following the administration of anti-TB drugs. This finding may be related to the higher incidence of TB in male populations. However, the precise relationship between gender and AT-DILI is still the source of much debate (Chang et al., 2008). Our present results were consistent with those reported previously by Kwok et al. (Chang et al., 2008); however, other studies failed to find any significant differences when comparing the incidence rate of AT-DILI between male and females (Yee et al., 2003; Gülbay et al., 2006). In addition, some studies have reported that the risk of AT-DILI in females is greater than that in men (Døssing et al., 1996; Devoto et al., 1997; Teleman et al., 2002) and that women may be more susceptible to certain drugs, such as minocycline and methyldopa, and are therefore more prone to chronic autoimmune hepatitis (Yu et al., 2017). Furthermore, our analysis also indicated that TB patients with higher incomes are more likely to develop AT-DILI. This may be due to a potential decline of immunity induced by the daily pressure/workload experienced by patients earning a high-income. We are the first to investigate the relationship between income and DILI; consequently, we are unable to compare our results with previous studies. Over recent years, the incidence of TB amongst the population of white-collar workers and high-income groups has increased in Nanshan District. Furthermore, Nanshan District included the employees of large and local enterprises in TB monitoring schemes in 2021; this data may provide useful information in future analyses.

Next, we investigated the relationships between TB drug prescriptions and the positive rate of AT-DILI. We found that patients taking the 2FDC-HRZE half/4FDC-HR regimen had the highest positive incidence of AT-DILI (56.03%); this was followed by those taking the 2FDC-HRZE/4FDC-HR regimen (31.11%); the lowest rate was observed in patients taking the 2FDC-HRZ + EMB/4FDC-HR regimen (24.27%). Importantly, the dosage of PZA in the first two schemes showing high positivity was 1,600 mg; this was higher than the usual dosage of 1,500 mg. Our current results suggest that the FDC regimen for anti-TB treatment has a higher risk of abnormal liver functioning or AT-DILI; it is likely that this finding was related to the high dosage of PZA. Studies have also shown that INH and PZA can cause serious damage to liver function; even after these drugs are terminated, AT-DILI can still develop (Ichai et al., 2010). The combined use of RFP and PZA for the treatment of TB can also cause severe AT-DILI; PZA appears to be the major contributor to such injury (Ridzon et al., 1997). In a survey of elderly TB patients in France, the use of PZA was found to be the only independent risk factor for adverse drug reactions (Rousset et al., 2020). Similar studies, conducted in Japan, found that the mortality rate of TB patients over 80 years-of-age was also positively associated with use of PZA (Hagiwara et al., 2019). Therefore, an excessive dosage of PZA in FDCs may be an important risk factor for the development of AT-DILI. Consequently, it is important that clinicians remain vigilant in clinical practice with regards to the development of AT-DILI in patients undergoing treatment for TB. Furthermore, murine studies have found that combined treatments, featuring INH/RFP, can cause the accumulation of endogenous hepatic toxin protoporphyrin IX (PPIX) or bile acids by downregulating the bile acid transporter; this process destroyed liver function and led to the development of AT-DILI (Guo et al., 2015). Because the INH/RFP combination is generally applied during the entire course of initial TB treatment as a standard strategy, the prospect of AT-DILI is unavoidable.

In the present study, we also found that as medication increased, there was a gradual increase in the positive incidence of AT-DILI or abnormal liver functioning in patients under various medication schemes. A previous study of 926 patients with TB who were followed-up for 4,122.9 persons × months (pm) in Taiwan showed that 12.0% of patients developed AT-DILI in the first 38 days after commencing treatment and that the positive incidences of AT-DILI in response to INH, RFP, and PZA, were 0.59, 0.69, and 3.71 per 100 pm, respectively (Shu et al., 2013). These results indicated that longer periods of medication use will lead to the gradual accumulation of drug metabolites in the liver, thus increasing the likelihood of more severe AT-DILI.

There are some limitations to this study that need to be considered. First, this was a retrospective study. The data were acquired from our hospital information system and were collected in real time. However, the data items were relatively limited. Some of the data required for the determination of RUCAM scores were not available. Consequently, we cannot confirm the specific causality of AT-DILI; furthermore, these our finding were only based on 287 positive patients. However, the purpose of this study was to investigate the risk factors for AT-DILI or abnormal liver functioning. Our team has developed an early warning model for AT-DILI or abnormal liver functioning during the first-line treatment of patients with TB and can be used to facilitate clinical practice (Zhong et al., 2021). We assess the risk of abnormal liver functioning or AT-DILI for all patient before anti-tuberculosis treatment, and intervene in advance for patients who may have a higher risk, such as adjusting the FDC program to a single drug combination regime, increasing liver protection drugs, strengthening the frequency of follow-up during treatment. We collect relevant data to compare the positive rates before and after implementation of our model. In the future, we will continue to study the causal relationship between risk factors and AT-DILI to facilitate clinical diagnosis and treatment.

## Conclusion

We investigated patients receiving first-line drug treatment for TB and identified male gender, high annual income, prescription regime, drug dosage, and treatment time, as significant risk factors for the development of AT-DILI or abnormal liver functioning. In addition to the development of new anti-TB drugs with less side effects, it is important that we establish an effective and evidence-based scheme and/or predictive model for AT-DILI based on existing prevalence data. We hope that our findings provide a strong body of evidence for clinicians to select appropriate anti-TB prescription regimes to reduce the risk of AT-DILI in patients with TB (Zhang, 2019).

## Data Availability

The original contributions presented in the study are included in the article/Supplementary Material, further inquiries can be directed to the corresponding authors.
